# Assessment of a high-fidelity mobile simulator for intrauterine contraception training in ambulatory reproductive health centres

**DOI:** 10.3402/jecme.v5.30416

**Published:** 2016-02-12

**Authors:** Laura E. Dodge, Michele R. Hacker, Sarah H. Averbach, Sara F. Voit, Maureen E. Paul

**Affiliations:** ^a^ Department of Obstetrics and Gynecology, Beth Israel Deaconess Medical Center, Boston, MA, USA; ^b^ Department of Obstetrics, Gynecology and Reproductive Biology, Harvard Medical School, Boston, MA, USA; ^c^ Department of Epidemiology, Harvard T.H. Chan School of Public Health, Boston, MA, USA; ^d^ Affiliates Risk Management Services, Inc, New York, NY, USA

**Keywords:** simulation, high-fidelity, gynaecology, intrauterine device insertion, training

## Abstract

Objectives. Little is known about the utility of simulation-based training in office gynaecology. The objective of this cross-sectional study was to evaluate the self-reported effectiveness and acceptability of the PelvicSim™ (VirtaMed), a high-fidelity mobile simulator, to train clinicians in intrauterine device (IUD) insertion. Methods. Clinicians at ambulatory healthcare centres participated in a PelvicSim IUD training programme and completed a self-administered survey. The survey assessed prior experience with IUD insertion, pre- and post-training competency and comfort and opinions regarding the acceptability of the PelvicSim. Results. The 237 participants were primarily female (97.5%) nurse practitioners (71.3%). Most had experience inserting the levonorgestrel LNG20 IUD and the copper T380A device, but only 4.1% had ever inserted the LNG14 IUD. For all three devices, participants felt more competent following training, with the most striking change reported for insertion of the LNG14 IUD. The majority of participants reported increased comfort with uterine sounding (57.7%), IUD insertion on a live patient (69.8%), and minimizing patient pain (72.8%) following training. Of the respondents, 89.6% reported the PelvicSim IUD insertion activities as “valuable” or “very valuable.” All participants would recommend the PelvicSim for IUD training, and nearly all (97.2%) reported that the PelvicSim was a better method to teach IUD insertion than the simple plastic models supplied by IUD manufacturers. Conclusions. These findings support the use of the PelvicSim for IUD training, though whether it is superior to traditional methods and improves patient outcomes requires evaluation.

## Introduction

Simulation provides the opportunity to learn new procedures and practise and maintain skills without putting patients at risk. In obstetrics and gynaecology, simulation-based training has been used most often to teach laparoscopic skills in the operating room and vaginal delivery skills and management of obstetrical emergencies during labour and delivery.^1^ Research has found that simulation-based training in these areas improves learners’ competence and confidence.^2^, (3) In obstetrics, simulation-based training with high-fidelity models has been shown to improve skills when compared to traditional teaching models because high-fidelity models provide more lifelike sensory feedback.^4^

Less is known about the application of simulation-based training in office gynaecology, including family planning procedures. This gap exists despite calls from the American Congress of Obstetricians and Gynecologists and other experts to increase training of diverse practitioners in the provision of long-acting reversible contraceptive methods, most notably the intrauterine device (IUD) and contraceptive implant.^5^–(7) Increased uptake of these highly effective contraceptive methods has the potential to decrease the rate of unintended pregnancy,(5,8–10) which has remained at approximately 50% in the United States for the past two decades.^11^ In recent studies, education and training in IUD insertion have been associated with increased likelihood of IUD provision in clinical practise.^6^, (7)(12) Limited research on IUD training using low-fidelity simulation-based models has demonstrated improvements in self-reported confidence and comfort following training, as well as decreased anxiety.^13^–(15)

**Figure 1:**
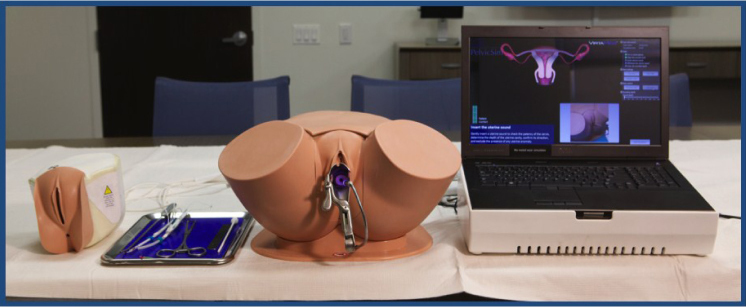
PelvicSim™ mobile simulator. Photo courtesy of Affiliates Risk Management Services, Inc.

Although IUD manufacturers provide simple, plastic low-fidelity models for practising IUD insertion, the PelvicSim™ (VirtaMed, Zurich, Switzerland) is the only high-fidelity virtual reality simulator currently marketed for this purpose. This mobile simulator consists of a pelvic model made of a realistic rubber-like material, several pelvic inserts (including anteverted and retroverted uteri and nulliparous and parous cervices), IUD training devices, and a laptop computer (Figure 1). It accommodates insertion of the following three types of IUDs currently approved by the US Food and Drug Administration: the copper T380A (ParaGard^®^, Teva Women's Health, Inc., North Wales, PA, USA) and the two levonorgestrel-releasing IUDs (LNG20, Mirena^®^, and LNG14, Skyla^®^, Bayer HealthCare Pharmaceuticals, Inc., Whippany, NJ, USA). Using sophisticated biomedical sensors, the simulator can measure each step of the insertion process. The insertion path is measured by sensors located within the simulated uterus that respond to sensors located in the uterine sounding device, which is used to measure the depth of the uterus before inserting the intrauterine contraceptive device. During placement, the learner has the option of guided or unguided insertions; guided insertions consist of visual and verbal cues including warnings and illustrations of the devices to demonstrate correct placement. A “comfort meter” is used to indicate manoeuvres that may elicit patient discomfort. Pain is represented by a visual scale that changes from green to yellow to red in response to clinician actions. Pain responses are provoked by excessive force, speed, or pressure during the insertion, and the pain lessens when the trainee alters his or her practise. The simulator provides feedback that is saved and can be reviewed later by both the learner and the instructor; this feedback includes video, audio, and discrete performance metrics delivered in real time.

In 2012, Affiliates Risk Management Services, Inc. (ARMS), the risk management services organization for a national network of reproductive health care affiliates in the United States, launched a novel mobile IUD training programme for affiliate clinical staff. Comprising 65 affiliates at the time of the study, this network provides reproductive health care training for approximately 3 million clients annually at 691 geographically dispersed health centres. This training programme was developed to introduce and/or improve IUD knowledge and skills among diverse practitioners with varying skill levels. The aim of this study was to evaluate the learner-assessed comfort, competency, and acceptability of the ARMS PelvicSim training programme.

## Methods

We conducted a study among clinical staff who participated in the IUD training programme from January 5, 2014 to November 6, 2014. This cross-sectional study assessed the one point in time when participants completed the training programme. Affiliate participation in training using the PelvicSim was voluntary. ARMS recruited affiliates through outreach at national meetings, a train-the-trainer conference, and individual calls. All clinical staff at each affiliate were invited to participate. Trainees were not included in this study. The PelvicSim was shipped via FedEx^®^ to each affiliate shortly before the scheduled training date.

**Figure 2:**
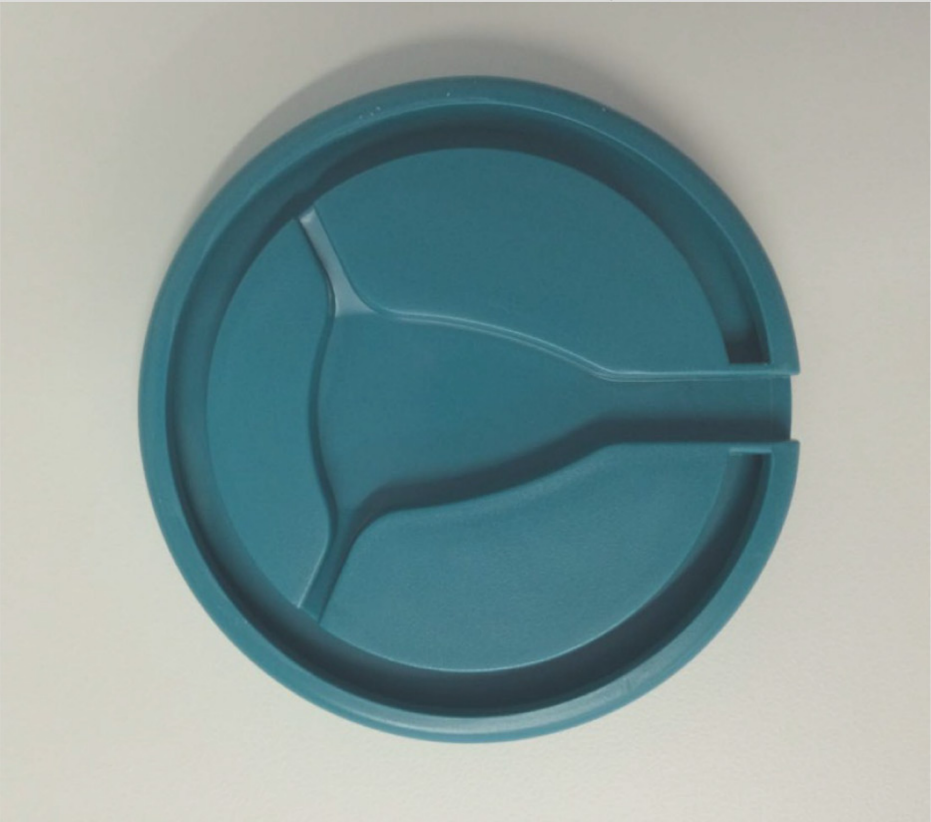
Plastic intrauterine device (IUD) training model provided by an IUD manufacturer (Bayer HealthCare Pharmaceuticals, Inc., Whippany, NJ, USA). Photo courtesy of Affiliates Risk Management Services, Inc.

One or two trainers from ARMS conducted each training programme, which consisted of an initial train-the-trainer session followed by a didactic session and hands-on practicum. The train-the-trainer session included instruction and practise on the assembly and disassembly of the simulator, as well as training on the operation of the simulators, including troubleshooting. The didactic session lasted 1 to 4 h; the content, determined by individual affiliate coordinators, may have included medical eligibility criteria for IUD use, client-centered counselling, case studies, complications management, and a demonstration of loading the Cu T380A device in the package. The hands-on practicum consisted of either individual trainings or group trainings. Clinicians earning continuing medical education (CME) credits were required to practise device insertion and were then assessed on sounding the uterus and inserting each device; these activities took a minimum of 45 min. For clinicians not earning CME credits, practicum trainings typically lasted 20 to 60 min, and although clinicians were encouraged to practise with all three types of IUDs, they may have chosen to focus on one or two devices. Group trainings involved two to four clinicians who took turns practising device insertion and observing their colleagues for a total of 60 to 90 min. Given that the training content was determined by individual affiliate coordinators and that training could be self-paced by the learner, participants spent varying amounts of time with the PelvicSim. Clinicians also were offered the opportunity to practise IUD insertion using a plastic model provided by an IUD manufacturer (Figure 2), which we considered “traditional” training, as well as the option to review videos on tenaculum placement and uterine sounding. Additional training included simulated practise of bimanual pelvic examinations. While the PelvicSim has the capacity to provide feedback on user performance, individual affiliate coordinators decided whether and how to assess their clinicians. Thus, there was no formal standardized debrief that all participants completed.

We distributed a self-administered, anonymous post-simulation questionnaire to clinicians who completed the PelvicSim IUD insertion training. The questionnaire assessed participants’ prior experience with IUD insertion, duration and components of their PelvicSim training, self-reported competency with each of the simulation-based tasks before and after training, change in comfort level with each of the tasks after training, and opinions regarding the acceptability of the PelvicSim alone and compared to the plastic low-fidelity models provided by the manufacturer. The questionnaire was pilot tested among clinicians with various levels of experience with IUD insertion and further refined based on feedback from these clinicians. The institutional review board at Beth Israel Deaconess Medical Center (BIDMC) designated this evaluation as being exempt from regulations governing human subjects’ protections.

### Statistical analysis

The study used a sample of convenience among all clinicians who participated in the evaluation component of the training session during the study period. Data are described as frequencies and proportions. Self-reported competency was assessed using a Likert scale (not at all competent = 0, competent with assistance = 1, independently competent for simple insertions = 2, and independently competent for all insertions = 3), and the change in self-reported competency before and after training was tested using the Wilcoxon signed rank test. We stratified experience with IUD insertion by whether the participant had placed at least 100 IUDs of any type. All data analysis was conducted using SAS 9.4 (SAS Institute, Cary, NC, USA).

## Results

A total of 237 participants from 18 affiliates completed the survey. The response rate was 80.1%. Of these respondents, nearly all (97.5%) were female, and most (71.3%) were nurse practitioners. Most had experience inserting the LNG20 (91.9% had previously inserted the device, and 45.3% had inserted more than 100 devices) and Copper T380A (89.4% had previously inserted the device, and 44.5% had inserted more than 100 devices). Only 4.1% of respondents had ever inserted the LNG14 device (Table 1).

**Table 1:** Participant characteristics at the time of training with the PelvicSim™.

Characteristic	All participants *N*=237 Number (%)

Years in practice	
< 5	79 (33.3)
5–10	52 (21.9)
11–15	27 (11.4)
16–20	18 (8.0)
> 20	61 (25.7)
Gender	
Female	231 (97.5)
Male	6 (2.5)
Type of provider	
Nurse practitioner	169 (71.3)
Physician assistant	28 (11.8)
Certified nurse midwife	24 (10.1)
Obstetrician–gynaecologist	5 (2.1)
Family medicine physician	4 (1.7)
Registered nurse	1 (0.4)
Other	6 (2.5)
Number of LNG20 (Mirena^®^) IUDs inserted in career	
0	19 (8.1)
1–10	27 (11.4)
11–50	39 (16.5)
51–100	44 (18.6)
> 100	107 (45.3)
Number of Copper T380A (ParaGard^®^) IUDs inserted in career	
0	25 (10.6)
1–10	19 (8.1)
11–50	43 (18.2)
51–100	44 (18.6)
> 100	105 (44.5)
Number of LNG14 (Skyla^®^) IUDs inserted in career	
0	189 (95.9)
1–10	4 (2.0)
11–50	3 (1.5)
51–100	0 (0.0)
> 100	1 (0.5)
Number of other IUDs* inserted in career	
1–10	4 (26.7)
11–50	9 (60.0)
51–100	0 (0.0)
> 100	2 (13.3)

*Responses included Copper 7, Lippe's loop, Medicines360 LNG, progesterone insert; calculated among respondents who reported inserting other intrauterine contraceptive devices.

**Figure 3:**
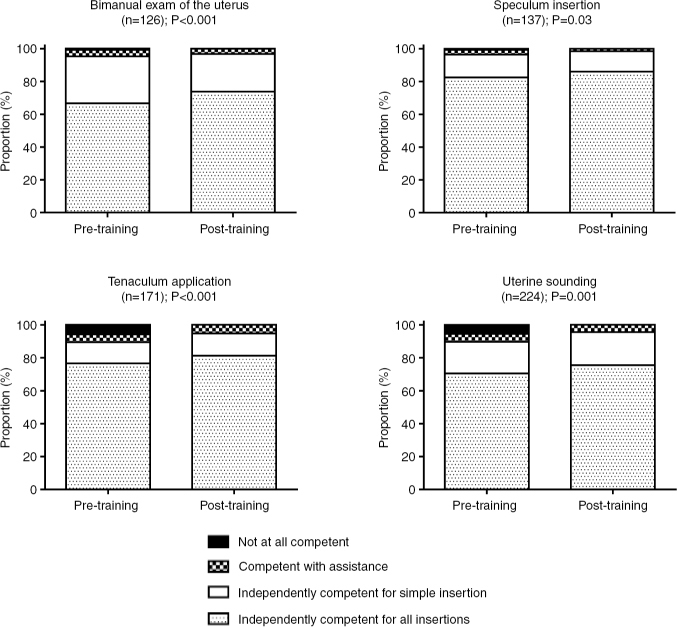
Self-reported competency before and after training with the PelvicSim among participants who utilized each training component. Competency calculated among respondents who reported both pre- and post-training competency and had used the individual training component.

**Figure 4:**
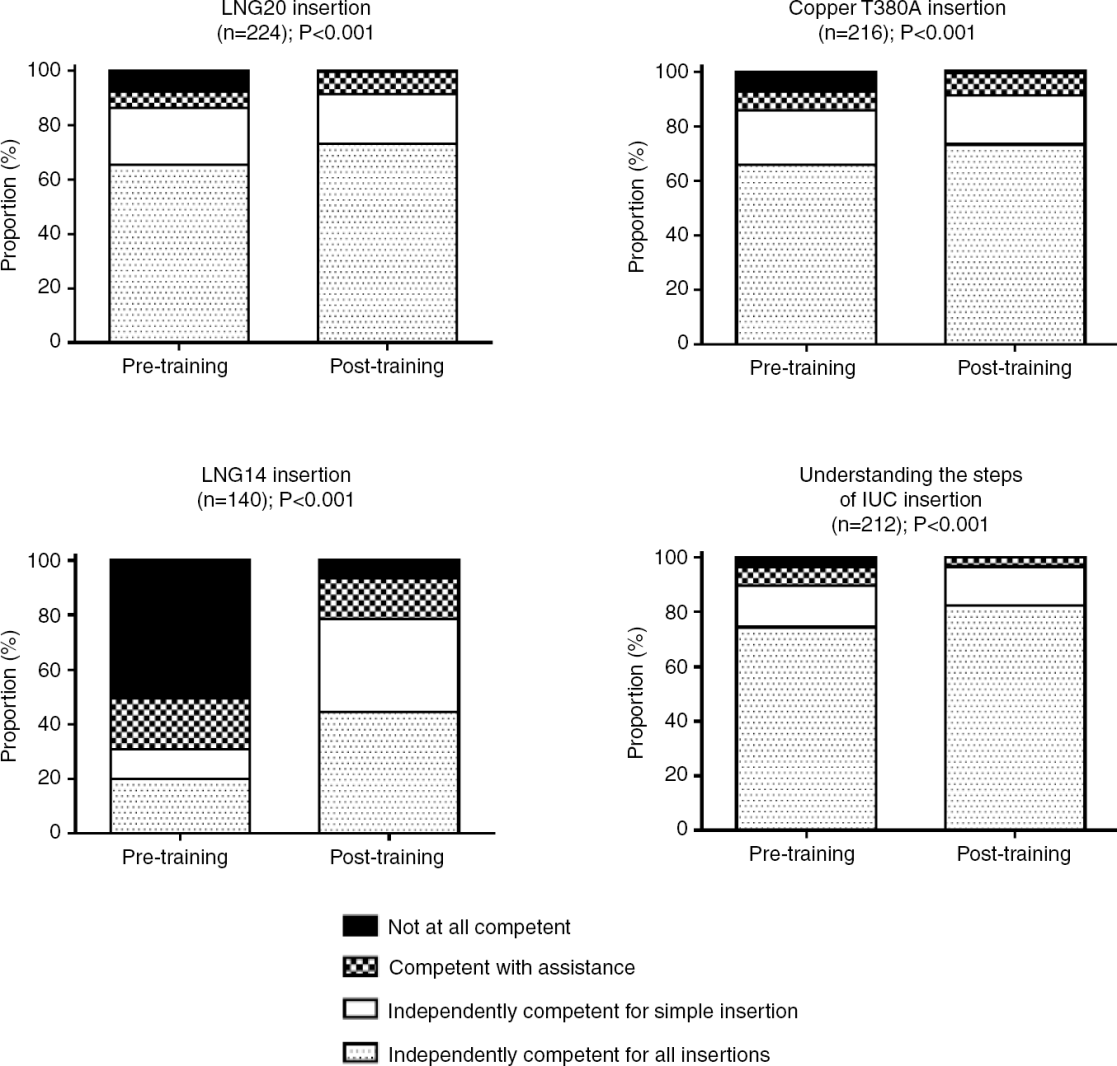
Self-reported competency before and after training with the PelvicSim among participants who utilized each IUD insertion component. Competency calculated among respondents who reported both pre- and post-training competency and had used the individual training component.

Among the respondents, 57.8% spent less than 1 h using the PelvicSim training components, and the vast majority (99.1%) spent fewer than 5 h. Regarding the tasks that they completed using the PelvicSim, more than half of respondents felt independently competent for all insertions prior to training, with the exception of LNG14 insertion, for which only 20.0% of participants felt independently competent for all insertions. Following training, self-reported competency increased for all tasks (all *p*<0.04; Figures 3 and 4), with the most striking change reported for insertion of the LNG14 (44.3% of participants felt independently competent for all LNG14 insertions after training). Despite the high level of perceived competency before training, the self-reported comfort level after training increased for all tasks (Table 2), with more than half of respondents feeling slightly or much more comfortable with uterine sounding (57.7%), understanding the steps of IUD placement (59.9%), IUD insertion on a live patient (69.8%), minimizing pain on a live patient (72.8%), LNG20 insertion (73.6%), Cu T380A insertion (74.1%), and LNG14 insertion (80.5%). Inexperienced users experienced significantly greater improvements in comfort for all tasks (all *p*≤0.001), with the exceptions of bimanual exam, speculum insertion, and LNG14 insertion.

**Table 2:** Self-reported change in comfort level following training with the PelvicSim.

Characteristic	Increased greatly Number (%)	Increased slightly Number (%)	Did not change Number (%)	Decreased slightly Number (%)	Decreased greatly Number (%)	P*

Bimanual exam						
Overall	14 (10.9)	21 (16.3)	94 (72.9)	0	0	0.73
< 100 IUD insertions	5 (8.5)	10 (17.0)	44 (74.6)	–	–	
≥ 100 IUD insertions	9 (12.9)	11 (15.7)	50 (71.4)	–	–	
Speculum insertion						
Overall	12 (8.6)	16 (11.5)	111 (79.9)	0	0	0.57
< 100 IUD insertions	5 (8.2)	9 (14.8)	47 (77.1)	–	–	
≥ 100 IUD insertions	7 (9.0)	7 (9.0)	64 (82.1)	–	–	
Tenaculum application						
Overall	27 (15.6)	37 (21.4)	109 (63.0)	0	0	0.001
< 100 IUD insertions	16 (23.2)	21 (30.4)	32 (46.4)	–	–	
≥ 100 IUD insertions	11 (10.6)	16 (15.4)	77 (74.0)	–	–	
Uterine sounding						
Overall	55 (24.2)	76 (33.5)	94 (41.4)	2 (0.9)	0	<0.001
< 100 IUD insertions	33 (39.3)	33 (39.3)	18 (21.4)	0 (0.0)	–	
≥ 100 IUD insertions	22 (15.4)	43 (30.1)	76 (53.2)	2 (1.4)	–	
LNG20 (Mirena) insertion						
Overall	84 (37.0)	83 (36.6)	55 (24.2)	4 (1.8)	1 (0.4)	<0.001
< 100 IUD insertions	46 (55.4)	27 (32.5)	9 (10.8)	0 (0.0)	1 (1.2)	
≥ 100 IUD insertions	38 (26.4)	56 (38.9)	46 (31.9)	4 (2.8)	0	
Copper T380A (ParaGard) insertion						
Overall	78 (35.5)	85 (38.6)	52 (23.6)	5 (2.3)	0	<0.001
< 100 IUD insertions	43 (53.1)	30 (37.0)	8 (9.9)	0	–	
≥ 100 IUD insertions	35 (25.2)	55 (39.6)	44 (31.7)	5 (3.6)	–	
LNG14 (Skyla) insertion						
Overall	94 (57.3)	38 (23.2)	30 (18.3)	2 (1.2)	0	0.36
< 100 IUD insertions	31 (57.4)	16 (29.6)	7 (13.0)	0	–	
≥ 100 IUD insertions	63 (57.3)	22 (20.0)	23 (20.9)	2 (1.8)	–	
Steps of placement						
Overall	63 (28.8)	68 (31.1)	87 (39.7)	1 (0.5)	0	<0.001
< 100 IUD insertions	36 (46.2)	24 (30.8)	18 (23.1)	0	–	
≥ 100 IUD insertions	27 (19.2)	44 (31.2)	69 (48.9)	1 (0.7)	–	
IUD insertion on a live patient						
Overall	45 (22.0)	98 (47.8)	59 (28.8)	3 (1.5)	0	<0.001
< 100 IUD insertions	21 (28.9)	42 (57.5)	9 (12.3)	1 (1.4)	–	
≥ 100 IUD insertions	24 (18.2)	56 (42.4)	50 (37.9)	2 (1.5)	–	
Minimizing pain on a live patient						
Overall	71 (32.1)	90 (40.7)	57 (25.8)	3 (1.4)	0	<0.001
< 100 IUD insertions	34 (43.0)	33 (41.8)	12 (15.2)	0	–	
≥ 100 IUD insertions	37 (26.1)	57 (40.1)	45 (31.7)	3 (2.1)	–	

Calculated among respondents who reported having used the individual training component.*Compares respondents who had performed <100 IUD insertions to those with ≥100 IUD insertions.

Participants rated video playback, photos of the insertion path, varying axis of the uterus, and IUD insertion activities as the most valuable components of the PelvicSim, with more than half of the participants rating each of these components as highly valuable. Bimanual examination of the uterus and the instructional videos were viewed as the least valuable (Table 3). There were 144 participants with previous experience using the traditional plastic models. Of these participants, 69.2% felt that the PelvicSim was much better for simulating IUD insertion on a live patient, and 82.6% expressed that it was much better as an overall method to teach IUD insertion (Table 4). Following the training, all participants reported that they would recommend the PelvicSim training to colleagues.

**Table 3:** Participant ratings of the PelvicSim training components.^a^

Characteristic	Very valuable Number (%)	Valuable Number (%)	Average value Number (%)	Limited value Number (%)	Not valuable Number (%)

IUD insertion activities	121 (52.6)	85 (37.0)	18 (7.8)	6 (2.6)	0
Video playback	128 (59.5)	68 (31.6)	14 (6.5)	5 (2.3)	0
Simulated patient comfort	112 (49.3)	88 (38.8)	20 (8.8)	7 (3.1)	0
Feedback metrics	112 (49.3)	88 (38.8)	20 (8.8)	7 (3.1)	0
Photos of insertion path	130 (58.6)	76 (34.2)	13 (5.9)	3 (1.4)	0
Varying axis of the uterus	105 (53.3)	72 (36.6)	14 (7.1)	6 (3.1)	0
Instructional videos	80 (45.2)	71 (40.1)	20 (11.3)	6 (3.4)	0
Bimanual exam of the uterus	40 (43.5)	31 (33.7)	10 (10.9)	7 (7.6)	4 (4.4)
Inserting IUD in a nulliparous patient	52 (47.7)	37 (33.9)	11 (10.1)	7 (6.4)	2 (1.8)

^a^Calculated among respondents who reported having used the individual training component.

**Table 4:** Comparison of the PelvicSim to the plastic manufacturer models.^a^

Characteristic	Much better Number (%)	A little bit better Number (%)	No difference Number (%)	A little bit worse Number (%)	Much worse Number (%)

Ease of use	100 (70.4)	27 (19.0)	6 (4.2)	8 (5.6)	1 (0.7)
Similarity to IUD insertion on a live patient	99 (69.2)	29 (20.3)	5 (3.5)	9 (6.3)	1 (0.7)
Overall – as a method to teach IUD insertion	119 (82.6)	21 (14.6)	2 (1.4)	2 (1.4)	0

^a^Calculated among respondents who had experience using the plastic manufacturer models.

## Discussion

This evaluation study demonstrated benefits from using a high-fidelity mobile simulator to teach IUD insertion to clinicians in geographically dispersed ambulatory health care settings. The participants, many of whom had prior experience with IUD provision, reported increased competency and comfort with IUD insertion after training on the PelvicSim. The change in the proportion of respondents feeling independently competent before and after training was larger for the LNG14 IUD than for the other devices, which is likely because the LNG14 IUD is newer to the US market. All participants reported that they would recommend the PelvicSim to colleagues as an IUD training tool, and most participants indicated that the PelvicSim was superior to simple plastic models for teaching IUD insertion.

Other studies have reported positive results of simulation-based training for IUD provision, although ours is the first to evaluate a high-fidelity mobile simulator for this purpose. At one US academic institution, Nitschmann et al. found that third-year medical students’ self-reported competency in IUD insertion increased significantly after training on the Gaumard Family Planning Educator^®^ (Gaumard Scientific, Miami, FL, USA), a low-fidelity desktop simulator.^13^ Another US study reported that participation of third-year medical students in a workshop using papayas as simulation-based models for practising intrauterine procedures significantly increased students’ self-assessed comfort levels in counselling patients about IUD insertions, as well as their knowledge-based test scores.^14^ In research conducted at a school of nursing and midwifery in Iran, 57 midwifery students were randomly allocated to traditional lectures versus simulation-based training using part-task trainers and simulated patients to teach all major aspects of IUD service provision. Following the training, the students provided counselling and placement of IUDs under supervision in the clinic setting. Self-reported comfort scores increased after the intervention in both groups, but the increase was significantly greater in the simulation-based training group. In addition, compared to the students allocated to traditional training, those in the simulation-based group demonstrated significantly decreased anxiety levels post-training.^15^

The strengths of our study include a relatively large sample size that allowed us to detect changes in self-assessed competency and comfort, as well as a high response rate. However, the design did not allow us to directly compare the benefits of high-fidelity simulation-based training to traditional teaching methods. Most of the participants had prior experience in IUD placement; thus, we cannot draw conclusions about the impact of this simulation-based training on novices. Nonetheless, the large improvement in self-reported competency associated with LNG14 insertion, a skill that was new to nearly all (95.9%) providers in this study, would suggest that the simulator is effective at teaching new skills. Additionally, the inclusion of experienced users allowed us to assess opinions comparing the high-fidelity simulation-based training to the traditional training. Because our outcome measures of competency and comfort were self-assessed in a simulated-based learning environment, the potential effect of the simulation on actual clinical skills is unknown. In addition, some aspects of the training, including its duration and the type of training, were not standardized, and thus there was variation in the simulation experience among participants.

## Conclusion

In summary, high-fidelity mobile simulation has the potential to be a useful and novel means of training clinicians in intrauterine procedures. Our study supports a growing body of evidence that suggests that simulation-based training carries substantial benefits for learners, but further research is needed to determine whether high-fidelity simulation-based training is superior to traditional methods of instruction and whether the benefits translate into improvements in clinical care. To further these goals, we are conducting a randomized trial to compare outcomes after training in IUD provision using the PelvicSim versus a traditional model.

## Authors’ contributions

LED contributed to study design, performed data analysis, and wrote the manuscript. MRH contributed to study design, performed data analysis, and critically edited the manuscript. SHA and SFV contributed to study design and critically edited the manuscript. MEP contributed to study design and wrote the manuscript.

## Conflict of interest and funding

LED, MRH, and SHA received salary support for the presented work from the research grant provided by ARMS to BIDMC. MEP is employed full-time by BIDMC, and ARMS provides financial compensation to the BIDMC Department of Obstetrics and Gynecology for a portion of her salary to support her role as physician director of Patient Safety and Quality at ARMS. SFV is employed full-time by ARMS, which funded this study. ARMS contributed to the development of the PelvicSim™ intrauterine contraceptive (IUC) module, and ARMS receives an applicable royalty for the sale of any simulator that contains the module. In addition, ARMS received credit towards purchase of a new PelvicSim simulator during the study period; a contract provision with VirtaMed stipulated such credit if VirtaMed sold at least five simulators incorporating the IUC module to any third party within a 60-day period between 15 December 2013 and 15 December 2014. This work was supported with a grant from ARMS, to BIDMC.
